# The Endocannabinoid System Drives Eosinophil Infiltration During Eosinophilic Esophagitis

**DOI:** 10.1016/j.jcmgh.2025.101515

**Published:** 2025-04-11

**Authors:** Eva Gruden, Melanie Kienzl, Laura Danner, David Markus Kaspret, Anja Pammer, Dusica Ristic, Oliver Kindler, Alfred D. Doyle, Benjamin L. Wright, Ulrike Taschler, Dominique Thomas, Robert Gurke, Franziska Baumann-Durchschein, Julia Konrad, Andreas Blesl, Hansjörg Schlager, Thomas Bärnthaler, Julia Kargl, Rudolf Schicho

**Affiliations:** 1 Division of Pharmacology, Otto Loewi Research Center, Medical University of Graz, Graz, Austria; 2 Division of Allergy, Asthma, and Clinical Immunology, Mayo Clinic Arizona, Scottsdale, Arizona; 3 Institute of Molecular Biosciences, University of Graz, Graz, Austria; 4 Institute of Clinical Pharmacology, Faculty of Medicine, Goethe University Frankfurt, Frankfurt am Main, Germany; 5 Fraunhofer Institute for Translational Medicine and Pharmacology (ITMP), Frankfurt am Main, Germany; 6 Fraunhofer Cluster of Excellence for Immune Mediated Diseases (CIMD), Frankfurt am Main, Germany; 7 Division of Gastroenterology and Hepatology, Department of Internal Medicine, Medical University of Graz, Graz, Austria

**Keywords:** Eosinophilic Esophagitis, Endocannabinoid System, Monoacylglycerol (Monoglyceride) Lipase, 2-Arachidonoylglycerol, Esophageal Epithelial Cells, Cannabinoid Receptors

## Abstract

**Background and Aims:**

Eosinophilic esophagitis (EoE) is a chronic, inflammatory, and antigen-driven disease of the esophagus. Total transcriptome data revealed alterations in the endocannabinoid system, in particular, down-regulation of monoacylglycerol lipase (MGL) in biopsies of patients with active EoE. We investigated the consequence of MGL down-regulation in mucosal biopsies of patients, and its implications for EoE development, such as recruitment of eosinophils.

**Methods:**

Levels of MGL substrate 2-arachidonoylglycerol, MGL enzyme activity, and MGL colocalization with epithelial cells were determined in mucosal esophageal biopsies of patients with EoE. Supernatant of human primary esophageal epithelial cells was used to determine eosinophil migration and activation. An inducible mouse model of EoE was used to test MGL inhibition and cannabinoid (CB) receptor antagonism in vivo.

**Results:**

MGL expression in esophageal epithelial cells from patients with active EoE is decreased, whereas 2-arachidonoylglycerol is increased compared with control subjects. Inhibition of MGL in epithelial cells leads to a proinflammatory phenotype capable of attracting eosinophils via CB_2_. Similarly, the EoE mouse model indicates that absence of MGL results in higher eosinophil infiltration. Targeting CB_2_ reduced the number of infiltrating eosinophils in the esophagi of mice.

**Conclusions:**

This study is the first of its kind to investigate the involvement of altered expression of endocannabinoid system components in EoE, and partly explains recent findings of more inflammatory features post EoE-treatment in cannabis users. Our findings could pave the way for research into alternative treatment options for EoE and call for caution regarding the use of cannabinoids in EoE.

## Graphical abstract

**Figure:**
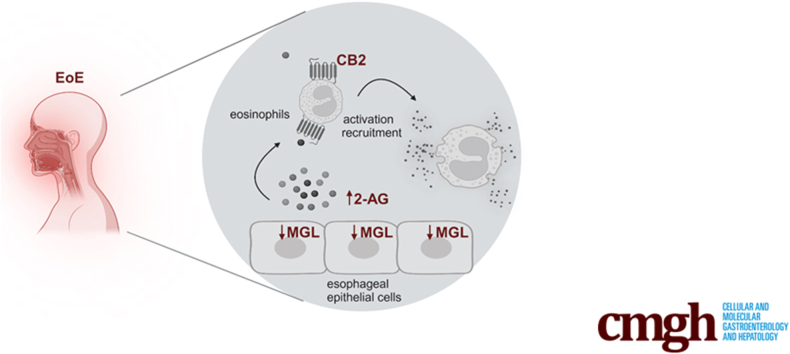

**Summary**

This paper shows downregulation of monoacylglycerol lipase and upregulation of 2-AG in epithelial cells of esophageal mucosal biopsies from active eosinophilic esophagitis (EoE) patients. Moreover, 2-AG recruits eosinophils via cannabinoid receptor 2 indicating involvememt of the endocannabinoid system in EoE disease.

Eosinophilic esophagitis (EoE) is an increasingly prevalent chronic disease of the esophagus characterized by T-helper type 2 (T_H_2) cell inflammation, resulting in eosinophilic infiltration.^1^ Extensive research has previously established the crucial role of esophageal epithelial dysfunction in disease development.^2^ The epithelium was also described as the target of the most prominent type 2 cytokine involved in EoE pathogenesis, interleukin (IL)13, which leads to barrier disruption, decreased differentiation, tissue remodeling, and eosinophil recruitment.^3^

The endocannabinoid system (ECS) is a network of receptors, endocannabinoids, such as 2-arachidonoylglycerol (2-AG), and enzymes that are involved in their synthesis and degradation (monoacylglycerol lipase [MGL]), and have long been considered pivotal in maintaining intestinal homeostasis and gut barrier integrity.^4^ MGL is a serine hydrolase that degrades monoglycerides, and its deletion or pharmacologic blockade results in higher levels of its substrate 2-AG.^5^^,^^6^ Thus, higher levels of 2-AG are bioavailable and capable of binding to its receptors, cannabinoid receptor 1 (CB_1_) or cannabinoid receptor 2 (CB_2_). In this way, MGL, 2-AG, and the cannabinoid receptors are components of an axis participating in many physiological and pathophysiological processes.^5^ In the central nervous system, the MGL-2-AG-CB_2_ axis acts as a functional entity, and attenuates stress-related mechanisms^7^ and fear-conditioned analgesia.^8^ Its effects in diseases of the human gastrointestinal tract, and the interplay of its components at the cellular level are, however, less known.

Because of the specific effects of (endo)cannabinoids on cytokine production, cell migration, T-cell responses, cell proliferation, and apoptosis, components of the ECS are recognized today as important drug targets in diseases characterized by an inflammatory response.^9^^,^^10^ In particular, previous studies showed that the activation of CB_2_ potentiates human eosinophil and group 2 innate lymphoid cell (ILC2) responsiveness, and aggravates allergic responses in models of allergic (T_H_2) lung inflammation.^11^^,^^12^ Additionally, we and others have extensively described the role of the ECS in gastrointestinal diseases and the esophagus.^9^^,^^13^^,^^14^ However, to date, data on the effects of (endo)cannabinoids, specifically in the context of allergic diseases, remain controversial.^15^^,^^16^

With the reported incidence and prevalence of cannabis use among the adult population reaching an all-time high,^17^ it has become essential to understand its effect on EoE pathology. It was shown that an increasing fraction of the population (approximately 3.9%) consumes *Cannabis sativa* or its products either for medicinal or recreational use daily, whereas increasing instances of cannabis allergy have been reported.^18^ Importantly, a review by Buckley et al ^19^ summarized the common use of cannabis products for pain management in patients with IBD. In line with this, Δ^9^-THC (the psychoactive compound found in cannabis) is known to act, next to CB_1_, also as a CB_2_ agonist,^4^ which could potentiate inflammation in EoE. A recent study by Borinsky et al ^20^ identified that cannabis users initially present with milder endoscopic findings, but also exhibit greater inflammatory features on endoscopy posttreatment. Therefore, there remains an unmet need to investigate the involvement of the ECS in allergy and EoE. Herein, we aimed to characterize alterations of ECS member expression in EoE. We identified decreased gene and protein expression levels of MGL in esophageal epithelial cells of patients with active EoE, which led to increased levels of the endocannabinoid 2-AG that has the potential to recruit and activate human or mouse eosinophils via its action on CB_2_. Importantly, we demonstrate that targeting CB_2_ in a preclinical model of EoE has the potential to decrease eosinophilic infiltration.

## Results

### MGL Expression is Reduced During Active EoE

**Figure 1:**
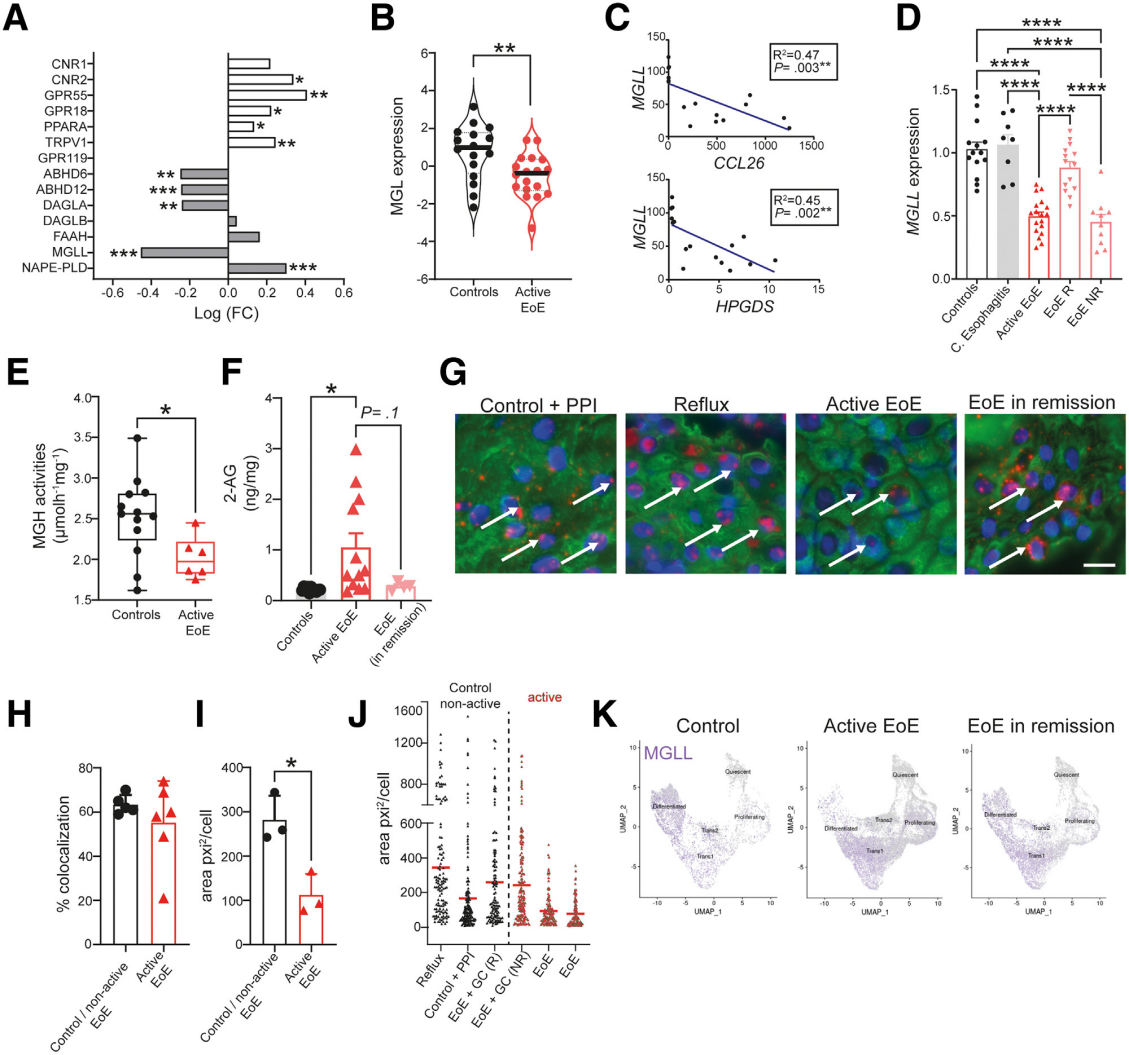
**MGL reduction in epithelial cells during active EoE leads to higher 2-AG levels.** (*A-D*) Publicly available datasets showing expression of ECS members. (*A*) Fold change of ECS gene expression and (*B*) protein expression of MGL in mucosal biopsies of patients with active EoE versus control subjects. (*C*) Correlation of MGLL (encodes MGL) with CCL26 (encodes eotaxin-3) or hematopoietic prostaglandin D_2_ synthase (HPGDS) in combined donor biopsies. (*D*) Gene expression levels of MGL in control subjects, chronic (*C*) esophagitis, and EoE patients, responsive (EoE R) or unresponsive (EoE NR) to corticosteroid treatment. (*E*) MGH activity measured as glycerol released from control and active EoE samples. (*F*) Levels of 2-AG in esophageal mucosal biopsies from patients with EoE and control subjects, as measured by liquid chromatography/mass spectrometry. Correlations were determined using Spearman correlation coefficient. (*G*) Representative micrographs of MGL mRNA ISH (*red*) colocalizing with cytokeratin-positive epithelial cells (*green*) in human esophageal mucosal biopsies (calibration bar: 20 μm). Cell nuclei labelled with DAPI (*blue*). (*H*) MGL mRNA colocalizing with epithelial cells. (*I*) Average area of MGL mRNA ISH signals/cell; mean + standard deviation, n = 3–6 sections/patient; 3 patients/group (active or nonactive EoE); all data evaluated by unpaired Student *t* test or 1-way analysis of variance. (*J*) ISH of MGL mRNA in human esophageal mucosal biopsies measured as area covered by MGL mRNA signals per cell (160–190 cells/patient; *dots* represent cells). Each column represents data from 1 representative individual patient. (*K*) UMAP analysis of different epithelial cell subsets expressing MGL mRNA (MGL gene: MGLL) acquired from EGID Express. Control + PPI, control subject on proton pump inhibitors; EoE + GC (R), patient with EoE responding to glucocorticoid treatment; EoE + GC (NR), patient with EoE not responding to glucocorticoid treatment. ∗*P* < .05, ∗∗*P* < .01, ∗∗∗*P* < .001, ∗∗∗∗*P* < .0001.

To assess potential changes in ECS expression levels, we first accessed publicly available total transcriptome datasets of active EoE and control patient biopsies (EGID express database).^21^ The genes related to the ECS (and other members of the "endocannabinoidome") and their differential fold-change of expression in active EoE are shown in Figure 1*A*. Most receptors considered part of the ECS were found to be differentially and significantly up-regulated in biopsies from patients with active EoE (Figure 1*A*). In contrast, enzymes of the ECS showed either up-regulation or down-regulation during active EoE (Figure 1*A*). The most prominent differentially regulated mRNA in active EoE was determined to be *MGLL*. Moreover, publicly available proteomic data confirmed reduced MGL transcript levels during active EoE inflammation (Figure 1*B*).^22^ The down-regulation of MGL showed a negative correlation with hematopoietic prostaglandin D_2_ synthase (which is reported to be increased during esophagus narrowing and in effector T_H_2 populations present in EoE) and a negative correlation with CCL26 (eotaxin-3) (Figure 1*C*).^23^, ^24^, ^25^

MGL reduction is a specific feature of acute EoE, but not of chronic esophagitis (Figure 1*D*).^26^^,^^27^ Finally, gene expression analysis indicated the recovery of MGLL expression in patients responding to corticosteroid treatment (Figure 1*D*; EoE R).^26^^,^^27^ We also performed monoglyceride hydrolase (MGH) activity assays and noted significant reduction in MGH activity in biopsies collected from patients with active EoE disease (Figure 1*E*). Importantly, reduced MGH activities correlated with the accumulation of 2-AG, and returned to normal levels (although not significantly) in patients that responded to corticosteroid treatment (EoE in remission) (Figure 1*F*).

### MGL Expression is Reduced in Epithelial Cells of Mucosal Biopsies from Patients with Active EoE

With previous studies highlighting the essential role of esophageal epithelium involvement in EoE pathology,^2^ we specifically investigated the expression levels of MGL mRNA in epithelial cells of the esophagus using an in situ hybridization (ISH) technique (Figure 1*G-J*). Representative images of MGL mRNA signals colocalized with cytokeratin-positive epithelial cells in esophageal mucosal biopsies from different patient groups are shown in Figure 1*G*. Although the percentage of epithelial cells colocalizing with MGL mRNA signals was not significantly altered in patients with active EoE versus control subjects (Figure 1*H*), the area covered by the MGL mRNA signals/cell was significantly reduced in the epithelial cells of active EoE mucosal biopsies (Figure 1*I*). We consistently observed smaller areas of MGL mRNA signals in epithelial cells from biopsies of patients with active EoE disease compared with the control and remission groups (Figure 1*J*). To pinpoint the subset of epithelial cells that could account for the loss of MGL mRNA signal, we analyzed publicly available single-cell sequencing datasets of esophageal epithelium in EoE (EGID Express). Rochman et al^28^ indicates a differentiation blockade or loss of differentiated cells in biopsies of active EoE disease. Moreover, we observed that differentiated cells represent a subset of epithelial cells with the highest MGL expression in control individuals (Figure 1*K*). Therefore, blockade or loss of differentiation in epithelial cells during active EoE could also contribute to the observed decrease in total MGL mRNA expression.

### Inhibition of MGL Expression and Activity in Human Primary Esophageal Epithelial Cells Leads to Increased Levels of Proinflammatory Mediators In Vitro

**Figure 2:**
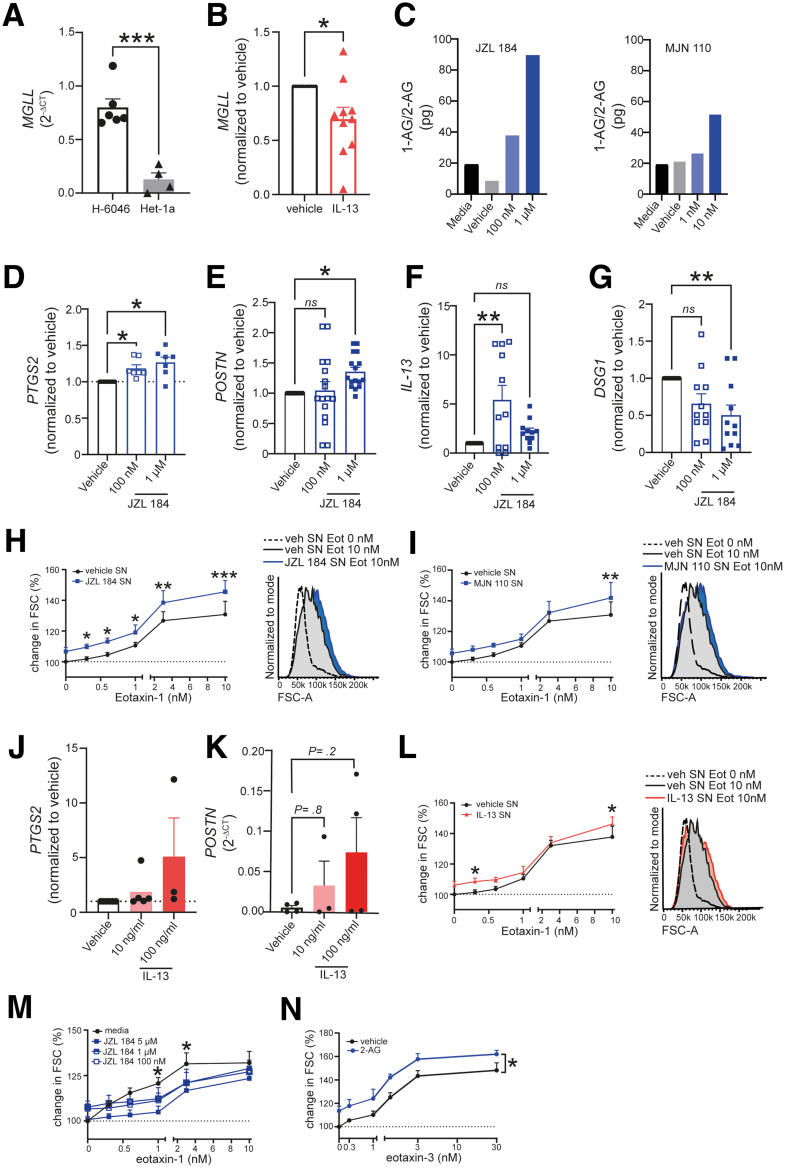
**In vitro inhibition of MGL leads to a proinflammatory phenotype in human primary esophageal epithelial cells (H-6046).** (*A*) MGL gene (MGLL) expression in primary esophageal epithelial cells (H-6046) and Het-1a cells. (*B*) Down-regulation of MGLL following 24 hours treatment with IL-13 (100 ng/mL) in H-6046 cells. n = 4-10. (*C*) Liquid chromatography/mass spectrometry measurements of 1-AG/2-AG in supernatants of epithelial cells treated for 24 hours with JZL 184 or MJN 110. (*D*) Gene expression of PTGS2 (encodes COX-2), (*E*) POSTN (encodes periostin), (*F*) IL-13, and (*G*) DSG1 (encodes desmoglein-1) following JZL 184 treatment for 24 hours; n = 7–14 cell passages of 3 different cell donors. (*H, I*) Size change of isolated human eosinophils following treatment with epithelial cell supernatants, and stimulation with eotaxin-1 (Eot). Representative histograms shown next to dose response curves. n = 5 eosinophil donors and supernatants of 5 different cell passages. (*J*) Gene expression of PTGS2 (COX-2) (normalized to vehicle control) and (*K*) POSTN (expressed as 2 ^-ΔCT^) following IL13 treatment for 24 hours; n = 3–5 cell passages of 3 different cell donors analyzed with 1-way analysis of variance. (*L*) Size change of isolated human eosinophils following treatment with epithelial cell supernatants (30 minutes RT) and stimulation with different concentrations of eotaxin-1. Representative FSC histograms of each experiment are shown next to eotaxin-1 dose response curves. n = 5 eosinophil donors and supernatants of 5 different cell passages. (*M*) Size change of isolated human eosinophils following treatment with JZL 184 in different concentrations (30 minutes, RT), and stimulation with different concentrations of eotaxin-1. n = 3 eosinophil donors. (*N*) Size change of isolated human eosinophils following treatment with 2-AG (1 μM, 30 minutes, RT), and stimulation with different concentrations of eotaxin-3. n = 3 eosinophil donors. Data analyzed by 1- or 2-way analysis of variance. ∗*P* < .05, ∗∗*P* < .01, ∗∗∗*P* < .001; Eot, eotaxin; FCS, forward scatter; ns, nonsignificant, SN, supernatant.

We next examined the potential outcomes of MGL inhibition in human primary esophageal epithelial cells. We first compared a commercially available immortalized esophageal epithelial cell line (Het-1a from ATCC) and epithelial cells isolated from human esophageal biopsies (H-6046 from Cell Biologics). We detected significantly higher MGL expression in the primary epithelial cells (Figure 2*A*). To mimic EoE conditions, we treated epithelial cells with IL-13 (100 ng/mL, 24 hours) and observed a significant decrease in MGL expression, compared with the vehicle-treated cells (Figure 2*B*). Similar observations of the down-regulation of MGL expression by IL-13 can be obtained from publicly available sequencing datasets of other esophageal epithelial cells, such as TE-7 and EPC2 (data not shown).^29^^,^^30^

Pharmacologic inhibition of MGL enzyme activity results in higher levels of 2-AG,^31^^,^^32^ which we also investigated in the supernatants of primary esophageal epithelial cells (H-6046). We observed that treating the cells with 2 different MGL inhibitors (MJN 110 and JZL 184) led to higher levels of 1-AG and 2-AG in supernatants (Figure 2*C*). It is known that 2-AG rapidly isomerizes to 1-AG at room temperature (RT), which is the more stable isomer. However, it is assumed that the release occurs only in the form of 2-AG. Therefore, the concentrations of 1-AG and 2-AG were evaluated as a sum parameter for this experiment. Moreover, MGL inhibition resulted in a shift toward proinflammatory markers, resulting in higher gene expression of PTGS2 (encoding COX-2), IL-13, and POSTN (encoding periostin) (Figure 2*D-F*). IL-13 and POSTN are both known to be increased during active EoE disease.^33^^,^^34^ In contrast, MGL inhibition downregulated the expression of DSG1 (encoding desmoglein-1), which is crucial for esophageal epithelial barrier integrity and has been proven to be down-regulated in EoE (Figure 2*G*).^34^

### Inhibition of MGL in Epithelial Cells Influences Eosinophil Activation

We next determined whether MGL inhibition in epithelial cells could influence eosinophil activation. For this purpose, we incubated isolated human eosinophils from healthy donors with the supernatants of epithelial cells that were pretreated either with vehicle or MGL inhibitors (JZL 184 or MJN 110), and we measured the activation of eosinophils in response to different concentrations of eotaxin-1 (Figure 2*H* and *I*). Our data show that short incubation (30 minutes, RT) of eosinophils with supernatants from MGL-inhibited epithelial cells potentiated eosinophil activation, as observed by greater size changes in flow cytometry (Figure 2*H* and *I*). Notably, we observed that IL-13 treatment dose-dependently induced gene expression (ie, an increase in POSTN and COX-2) and activation changes similar to pharmacologic MGL inhibition (Figure 2*J-L*). In contrast, direct treatment of eosinophils with JZL 184 dose-dependently decreased their activation in response to eotaxin-1 (Figure 2*M*). When eosinophils were directly incubated with 2-AG, we could observe a priming effect toward stimulation with eotaxin-3 (Figure 2*N*).

### 2-AG Participates in Eosinophil Recruitment During EoE Via Action on CB_2_

**Figure 3:**
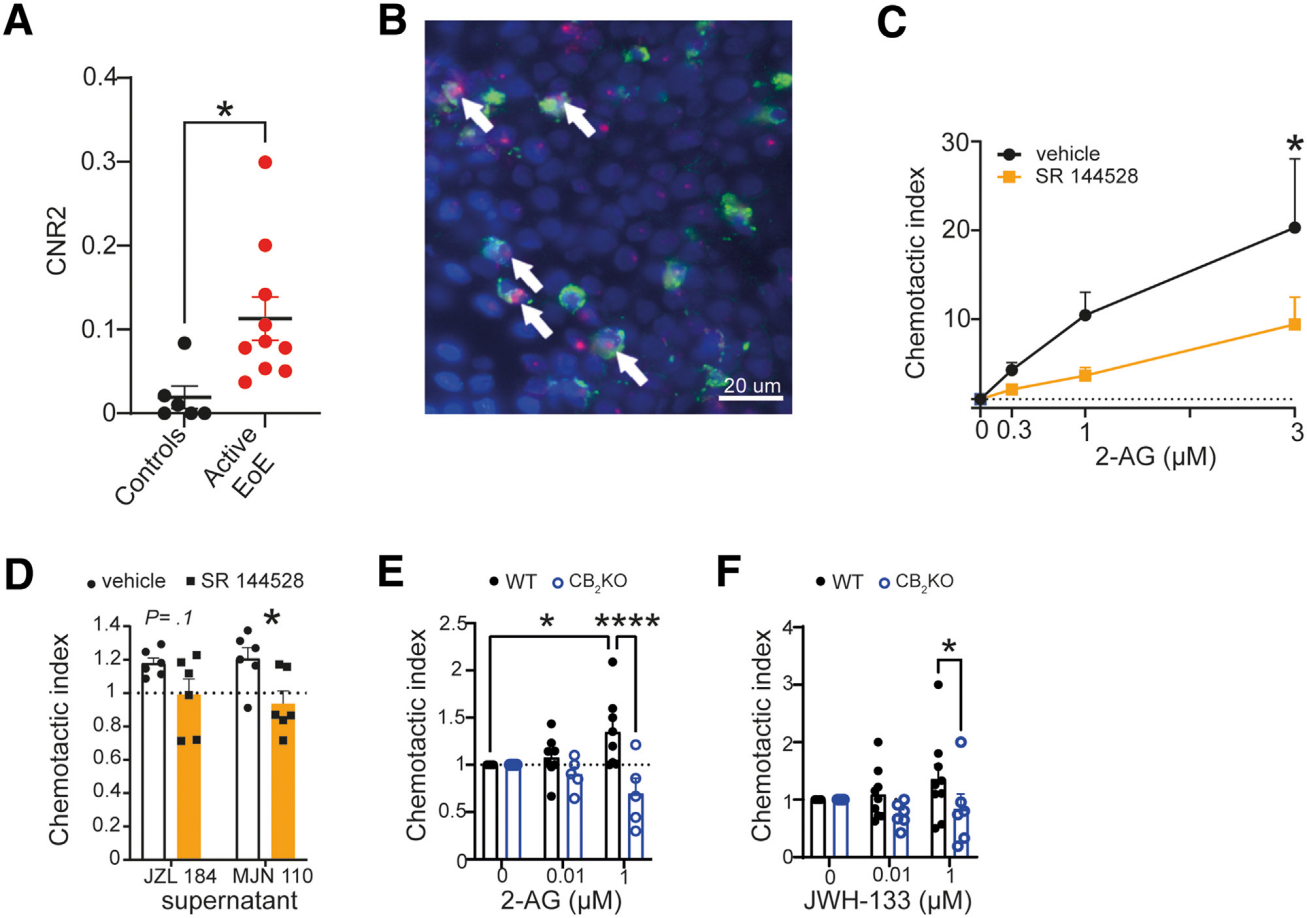
**CB_2_ is expressed in eosinophils of mucosal biopsies from patients with EoE and plays a role in migration.** (*A*) Expression of CNR2 (encodes CB_2_) acquired from total transcriptome analysis of esophageal mucosal biopsies by Sherrill et al.^21^ (*B*) ISH for CB_2_ mRNA (*red*) in EPX-positive cells (*green*) in sections of a mucosal esophageal biopsy from a patient with active EoE. Cell nuclei stained with DAPI (*blue*). (*C*) Migration of isolated human eosinophils, pretreated with vehicle (1 μM EtOH) or SR 144528 (1 μM), toward different 2-AG concentrations. (*D*) Migration of human eosinophils (pretreated with vehicle or SR 144528 [1 μM]) toward the supernatants of epithelial cells treated with JZL 184 (1 μM) or MJN 110 (100 nM). (*E, F*) Migration of splenocytes from WT or CB_2_ KO mice toward different concentrations of 2-AG (*E*) or JWH-133 (*F*). The number of migrated cells was determined by flow cytometry, and a chemotactic index was calculated as fold change of migrated cells, compared with vehicle control (no chemoattractant). n = 5–6 human eosinophil donors or 5–8 mice; means + standard error of means; 2-way analysis of variance. ∗*P* < .05, ∗∗∗∗*P* < .0001.

Because we discovered an increase in 2-AG in the mucosal biopsies of patients with active EoE (Figure 1*F*), we continued to study its action on its receptors. CB_1_ is not significantly altered in EoE (Figure 1*A*; CNR1: gene encoding CB_1_), whereas CNR2 (encodes CB_2_) gene expression is significantly increased in mucosal biopsies of patients with active EoE (Figures 1*A* and 3*A*). We, therefore, performed ISH for CB_2_ mRNA combined with immunofluorescence for EPX in sections of patient mucosal biopsies (Figure 3*B*). The representative staining indicates the positive colocalization of CB_2_ with eosinophils in situ (Figure 3*B*).

We next used the specific CB_2_ inverse agonist SR 144528^35^ to test whether CB_2_ activation plays a role in human eosinophil migration toward 2-AG. We observed that isolated human eosinophils dose-dependently migrated toward 2-AG, and short pretreatment with SR 144528 significantly inhibited this migration (Figure 3*C*). Moreover, by testing the migration of eosinophils toward supernatants of epithelial cells treated with the MGL inhibitors JZL 184 and MJN 110, we observed that SR 144528 also reduced the number of migrated eosinophils (Figure 3*D*). To test the involvement of the CB_2_ receptor in mouse eosinophil migration, we isolated splenocytes of wild-type (WT) and CB_2_ knockout (KO) mice and stained them with a panel of antibodies (Supplementary Table 1). We then enumerated the chemotactic index of the gated eosinophils toward vehicle or increasing 2-AG concentrations. Our data show that WT mouse eosinophils migrate toward 2-AG and that this migration is impaired in eosinophils lacking CB_2_ receptors (Figure 3*E*). The migration of WT and CB_2_ KO mouse eosinophils toward a specific CB_2_ agonist (JWH 133) is shown in Figure 3*F*.

### An Inducible Mouse Model of EoE Recapitulates MGL Reduction in Epithelial Cells and the CB_2_ Increase Observed in Human Patients

**Figure 4:**
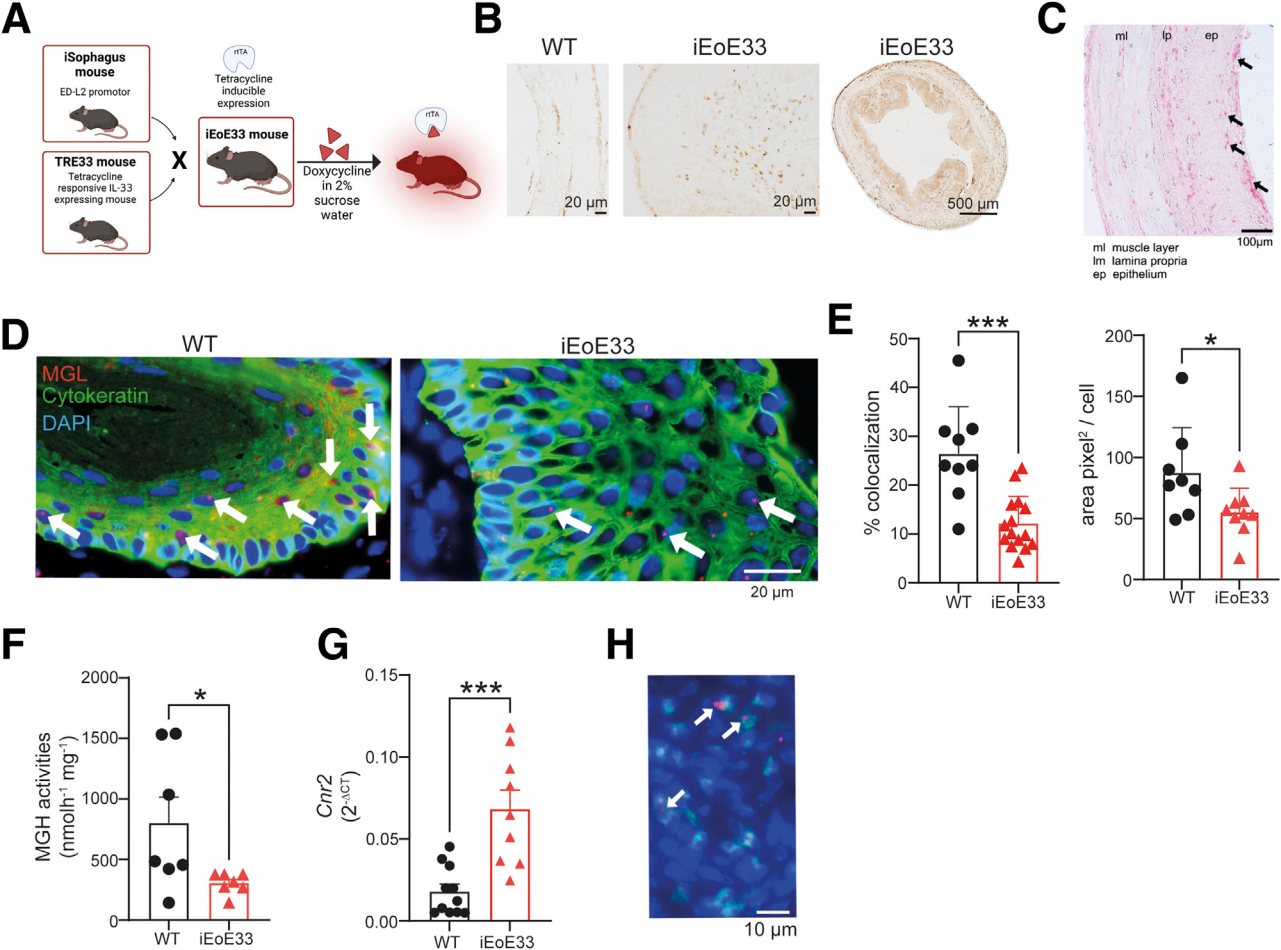
**An inducible mouse model of EoE (iEoE33) exhibits lower MGL expression in epithelial cells, and a higher total CB_2_ expression.** (*A*) A schematic representation of iEoE33 mouse design with a tetracycline-responsive, secreted and active IL-33 located under ED-L2 promotor, targeted to esophageal epithelium. (*B*) EPX immunohistochemical staining in esophageal sections of control (WT) and iEoE33 mice. (*C*) Sirius red staining of iEoE33 esophagus following doxycycline exposure. *Black arrows* indicate eosinophil infiltration in the epithelial layer. (*D*) Representative micrographs of MGL mRNA ISH (*red*) in cytokeratin positive epithelial cells (*green*) in WT and iEoE33 esophagi. (*E*) Quantification of MGL signals colocalizing with cytokeratin positive cells (*arrows*), and of area covered by MGL signals per cell. 3–4 sections evaluated from 2–4 mice per group. (*F*) MGH enzyme in esophageal sections of WT and iEoE33 mice. n = 7 mice per group. (*G*) Cnr2 (encodes CB_2_) gene expression in WT and iEoE33 esophagus sections. n = 9–11 mice per group. (*H*) ISH for CB_2_ mRNA (*red*) in EPX-positive cells (*green*) in esophageal mucosa of iEoE33 mice. Nuclei are in *blue*. Means + standard deviation or standard error of means; unpaired Student *t* test. ∗*P* < .05, ∗∗∗*P* < .001.

Having confirmed alterations in ECS levels in human patients, we set out to validate and investigate these findings in a novel inducible mouse model of EoE.^36^ The model design is presented in Figure 4*A*. In brief, iEoE33 mice possess tissue-specific (ED-L2 targeting promotor) inducible expression of secreted and active IL-33. On exposure to doxycycline in water (1 mg/mL in 2% sucrose), mice start exhibiting symptoms of EoE, such as pain and weight loss. On esophagus extraction, tissue thickening and increased immune cell infiltration can be observed.^36^, ^37^, ^38^, ^39^ The increase in IL-33 expression in iEoE33 esophagi also results in increased eosinophil infiltration, as shown by EPX staining in Figure 4*B* or Sirius red staining (Figure 4*C*), as reported previously.^36^^,^^39^ Our MGL ISH analysis of cytokeratin-stained cells in iEoE33 esophagi revealed reduced MGL mRNA signals in epithelial cells (Figure 4*D* and *E*). This reduction in MGL mRNA expression was reflected by reduced MGH activity in iEoE33 versus WT esophagi (Figure 4*F*), comparable with the human condition. Finally, similar to human EoE, iEoE33 mice showed higher CB_2_ mRNA expression (Figure 4*G*), when compared with WT esophagi. We could also show colocalization of CB_2_ receptor mRNA with EPX-positive cells in mouse esophageal mucosa by ISH (Figure 4*H*).

### Absence or Inhibition of MGL Activity Exacerbates Eosinophil Infiltration in WT and iEoE33 Mice

**Figure 5:**
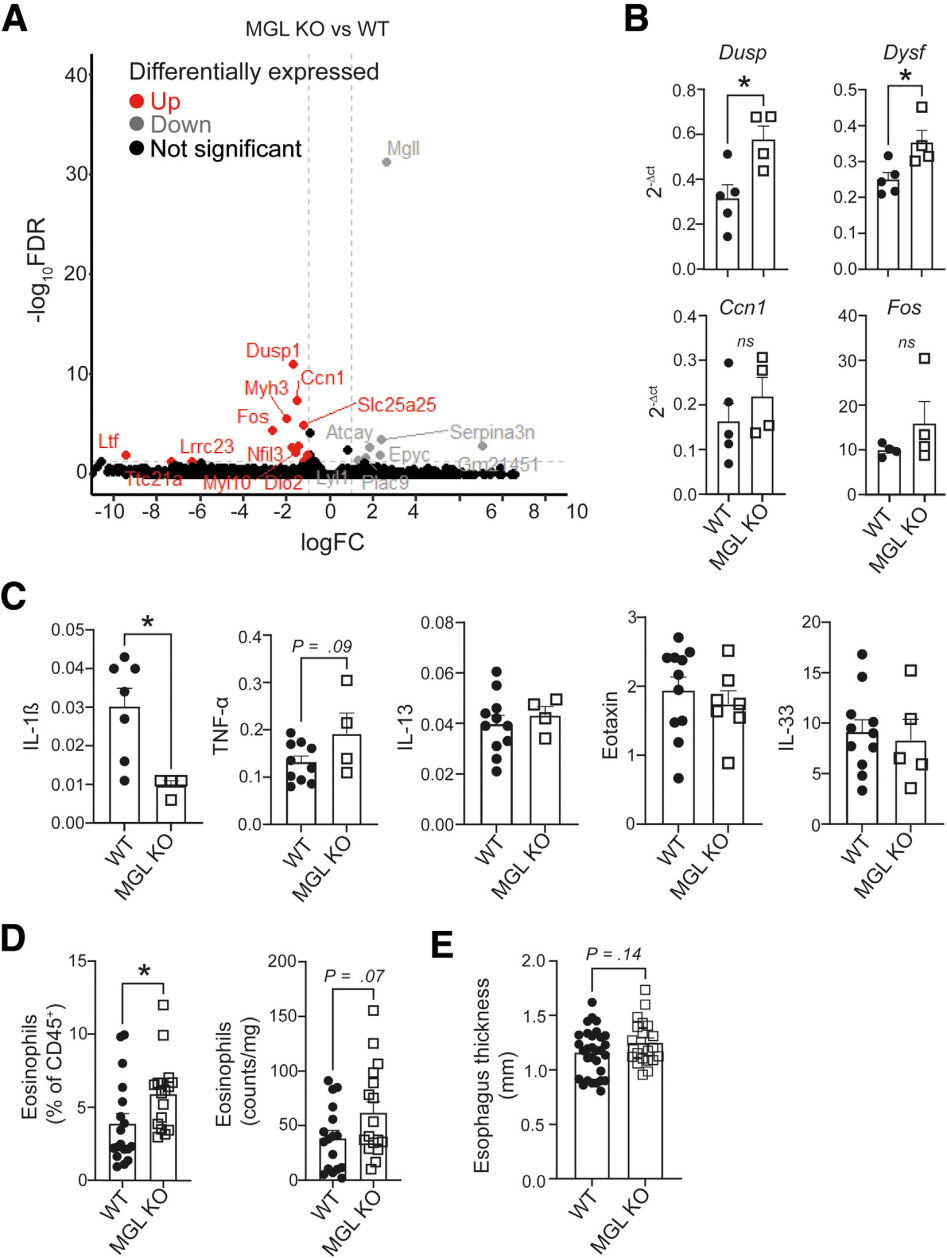
**Absence of MGL exacerbates eosinophil infiltration in healthy and iEoE33 mice.** (*A*) RNAseq of differentially expressed genes in MGL KO versus WT mouse esophagi (n = 4). In total, 27 genes were differentially regulated in MGL KO esophagi (fdr <0.05). (*B*) Validation of Dusp, Dysf, Ccn1, and Fos expression by quantitative polymerase chain reaction in WT versus MGL KO esophagi (n = 4). (*C*) Multiplex enzyme-linked immunosorbent assay measurements of cytokine concentrations in snap-frozen esophagus segments expressed in pg per mg of tissue (n = 4–7). (*D*) Flow cytometric determination of eosinophil percentage and numbers in single cell suspensions of healthy mouse esophagi (eosinophils identified according to Supplementary Table 3 and Figure 6*A*). (*E*) Combined data of esophagus thickness measurements of healthy WT and MGL KO mice. Esophagi were extracted from male and female 10- to 14-week-old mice and thickness was measured with a caliper by 2 blinded investigators. Average thickness measurement per esophagus was calculated from the measurements and presented on the graph. n = 20–30; data are shown as means + standard error of means and is analyzed with unpaired Student *t* test. ∗*P* < .05. TNF, tumor necrosis factor.

Having shown a reduction in MGL expression during active EoE disease, we investigated whether MGL down-regulation was a consequence of an inflammatory environment or whether MGL downregulation by itself could promote EoE development. For this reason, we performed a total transcriptome analysis of mRNA extracted from the esophagi of healthy WT and MGL KO mice (Figure 5*A*). RNAseq revealed multiple significantly up-regulated genes associated with inflammation in MGL KO esophagi, such as Dysf, Dusp, Ccn1, and Fos (Figure 5*B*).^40^ A table of significantly (fdr <0.05) differentially regulated genes in MGL KO versus WT esophagi can be found in Supplementary Table 2. Up-regulation of Dusp and Dysf was later validated by quantitative polymerase chain reaction (Figure 5*B*). We followed our analysis by performing a custom multiplex enzyme-linked immunosorbent assay to determine whether these alterations in gene expression could result in a significant shift toward a type 2 inflammatory environment. We observed significantly lower levels of IL-1β in MGL KO esophagi and trends toward higher tumor necrosis factor (TNF)-α levels. However, the concentrations of EoE-relevant major type 2 cytokines, such as IL-13, eotaxin, and IL-33, were not altered in healthy MGL KO esophagi (Figure 5*C*). Because absence of MGL in mouse tissue has been previously reported to result in higher 2-AG levels,^31^ we next investigated if in our experiments, absence of MGL could lead to higher eosinophil numbers in esophagi. By conducting flow cytometry analysis, we could indeed observe increased numbers of eosinophils in whole MGL KO esophagi, either evaluated as percentage or as counts (Figure 5*D*). Despite potentially increased numbers of eosinophils in MGL KO esophagi, these mice did not show clinical signs of EoE (eg, thickened esophagus; Figure 5*E*).

**Figure 6:**
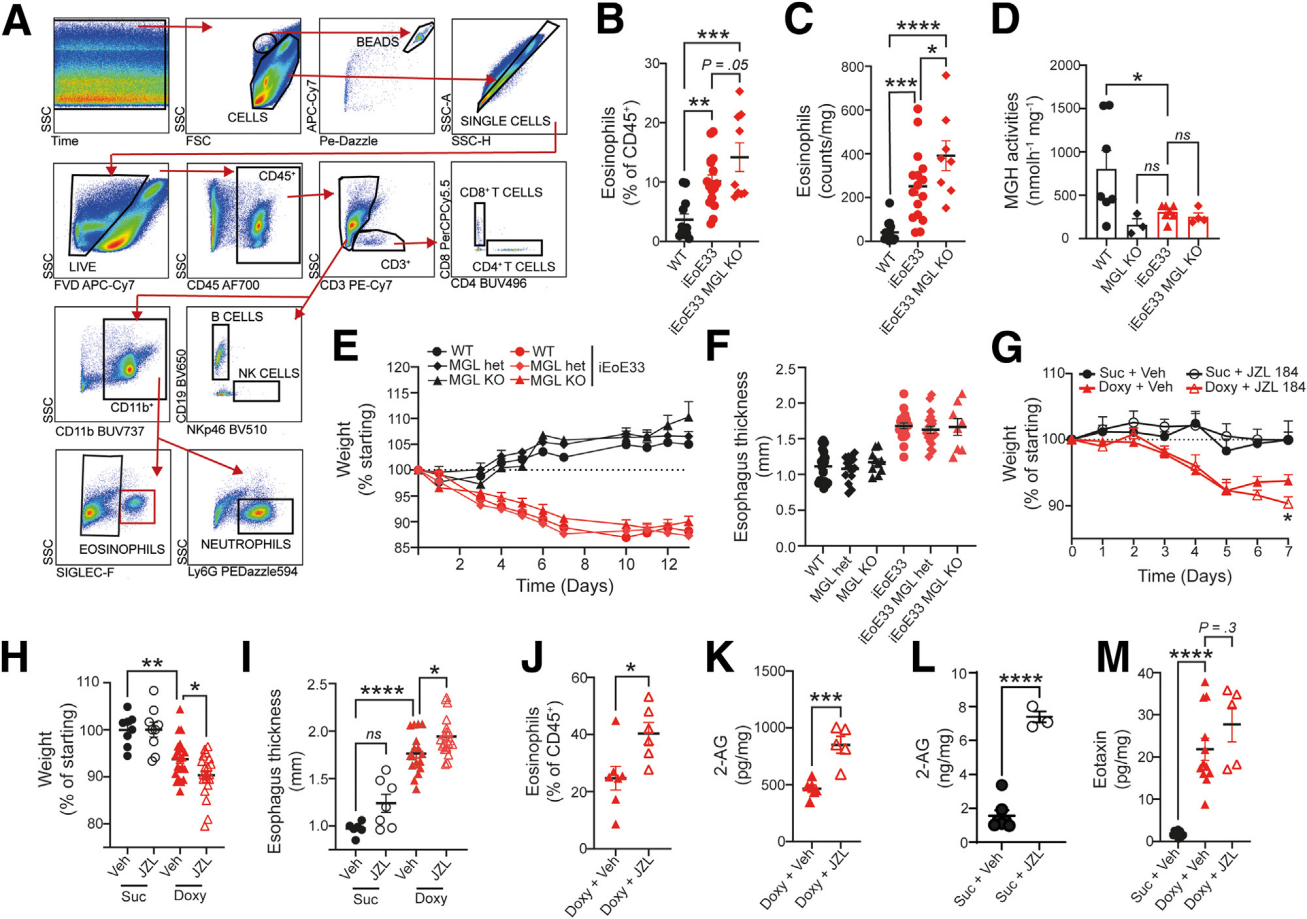
**Absence of MGL exacerbates eosinophil infiltration in iEoE33 mice.** (*A*) Gating strategy for determining immune cell populations in mouse esophagi. (*B-F*) iEoE33 mice were crossed with MGL KO mice, and exposed to doxycycline (1 mg/mL in 2% sucrose). (*B, C*) Flow cytometric determination of eosinophil percentage (*B*) and counts (*C*) in single cell suspensions of processed esophagi following doxycycline exposure. (*D*) MGH activity in mouse esophagi. (*E*) Weight loss of WT, MGL heterozygotes (het), and MGL KO either as WT or crossed with iEoE33 mice under doxycycline exposure. (*F*) Average esophagus thickness measurements of mice following 14 days of doxycycline exposure. (*G-M*) iEoE33 mice were treated with vehicle (Veh) or JZL 184 (16 mg/kg/d) for 7 days with either doxycycline (1 mg/mL in 2% sucrose [suc]) or sucrose alone (2%) exposure. (*G*) Weight tracking of mice during the 7-day sucrose alone or doxycycline exposure. (*H*) Weight loss (expressed as percent of starting weight on Day 7 of the experiment) and (*I*) esophageal thickness. (*J*) Infiltration of eosinophils in esophagi of vehicle- or JZL 184-treated iEoE33 mice. (*K, L*) 2-AG levels in extracted esophagi of vehicle (Veh) or JZL 184-treated (16 mg/kg/d) WT mice under 14 days of doxycycline (*K*) or sucrose (*L*) exposure as measured with mass spectrometry. (*M*) Eotaxin levels in extracted esophagi of vehicle (Veh)- or JZL 184-treated iEoE33 mice. n = 3–20; means + or ± standard error of means; unpaired Student *t* test or 2- or 1-way analysis of variance. ∗*P* < .05, ∗∗*P* < .01, ∗∗∗*P* < .001, ∗∗∗∗*P* < .0001.

To determine whether EoE-like pathology, including eosinophil infiltration, could be exacerbated by additionally lowering MGL expression or activity, we first crossed iEoE33 mice with MGL KO mice to generate iEoE33 MGL KO mice. These mice showed heightened eosinophil infiltration, as determined either as a percentage of CD45^+^ cells, or as counts measured by flow cytometry in the esophagi, following doxycycline exposure (Figure 6*A-C*). See antibody panel for eosinophil determination and gating strategy in Supplementary Table 3 and Figure 6*A*. Surprisingly, iEoE33 MGL KO mice did not exhibit lower MGH activity than MGL KO mice, most likely because MGH activity had already reached baseline levels in iEoE33 mice (Figure 6*D*). Consequently, there were no differences in EoE-like symptoms, such as weight loss and esophageal thickness, between iEoE33 MGL KO and iEoE33 mice (Figure 6*E* and *F*).

We, therefore, inhibited MGL activity in iEoE33 mice pharmacologically using JZL 184. We specifically chose to explore a short-term 7-day exposure to doxycycline, to identify early eosinophil infiltration. On Day 7 of doxycycline exposure, Pyon et al ^36^ reported the first significant thickening of the esophagus and infiltration of eosinophils into the epithelium and stroma in mouse esophagi. At the end of the experimental period, we detected significantly higher weight loss in the JZL 184–treated and doxycycline-exposed iEoE33 experimental group, when compared with vehicle control (Figure 6*G* and *H*). Moreover, esophagi of JZL 184–treated mice were significantly thicker compared with vehicle-treated and doxycycline-exposed mice (Figure 6*I*). We additionally observed higher eosinophil infiltration in the esophagi of JZL 184–treated mice (Figure 6*J*), which was accompanied by significantly higher 2-AG levels, further proving that JZL 184 intraperitoneal application is sufficient to inhibit MGL activity in esophagi (Figure 6*K*). This increase in 2-AG was even more evident in untreated WT animals not receiving doxycycline (Figure 6*L*). Finally, we did observe increasing eotaxin concentrations post–JZL 184 treatment in the esophagi (Figure *M*).

### Antagonism of CB_2_ Receptor Reduces the Number of infiltrated Eosinophils But Does Not Ameliorate Disease Pathology in a Mouse Model of EoE

**Figure 7:**
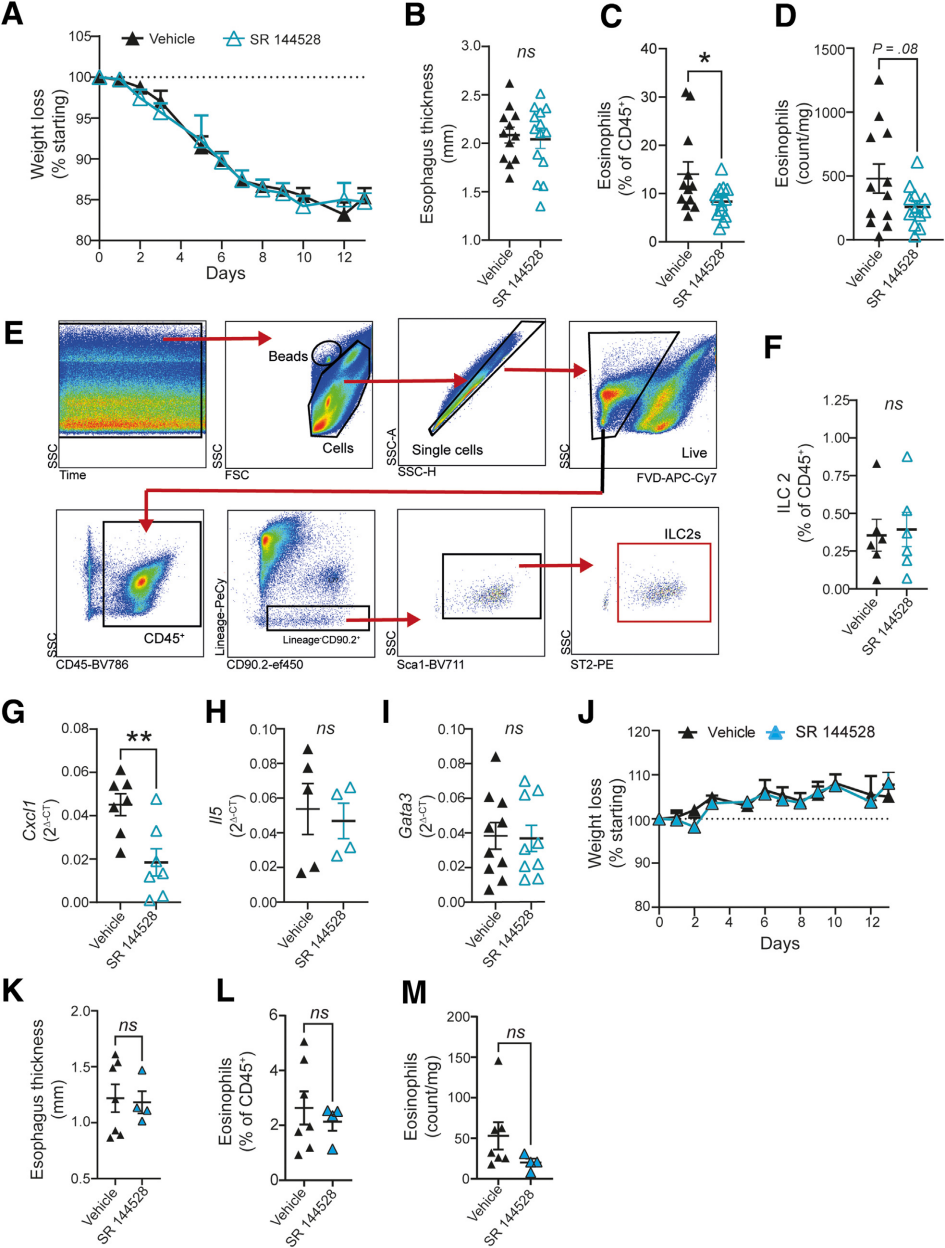
**Antagonism of CB_2_ decreases numbers of infiltrating eosinophils in the iEoE33 esophagus, but does not reduce disease pathology.** (*A-I*) iEoE33 mice were treated with vehicle or SR 144528 (10 mg/kg/d) for 14 days of doxycycline exposure (1 mg/mL in 2% sucrose). (*A*) Weight tracking and (*B*) esophageal thickness of treatment groups. (*C, D*) Number of eosinophils in esophagi of vehicle- or SR 144528-treated iEoE33 mice under doxycycline exposure evaluated by flow cytometry (according to Supplementary Table 3 and Figure 6*A*), (*C*) expressed as percent of CD45^+^, or (*D*) as counts per milligram. (*E*) Gating strategy for ILC2 determination in single cell suspensions of mouse esophagi. A minimum of 10 mg of iEoE33 esophagi are needed for accurate ILC2 determination. Antibody panel is listed in Supplementary Table 6. Counting beads were gated according to size (high SSC) and high double intensity in APC-Cy7 and PE Dazzle channels. ILC2 cells were determined as live, CD45^+^/CD90.2^+^Lineage^-^; Sca1^+^, ST2 receptor^+^ cells. (*F*) Percentage of ILC2s expressed as percent of all CD45^+^ cells in esophagi of treated iEoE33 mice evaluated by flow cytometry. (*G-I*) Quantitative polymerase chain reaction of inflammatory cytokines and markers in esophagi of treated iEoE33 mice following 14 days of doxycycline exposure. (*J-M*) WT mice were treated with vehicle or SR 144528 (10 mg/kg/d) for 14 days of doxycycline exposure (1 mg/mL in 2% sucrose). (*J*) Weight tracking and (*K*) esophageal thickness of WT treatment groups. (*L, M*) Number of eosinophils in esophagi of vehicle- or SR 144528-treated iEoE33 mice under doxycycline exposure evaluated by flow cytometry (*L*) expressed as percent of CD45^+^, or (*M*) as counts per mg. n = 4–14; means + or ± standard error of means; data evaluation by unpaired Student *t* test or 2-way analysis of variance. ∗*P* < .05, ∗∗ *P* < .01; ns, nonsignificant.

Because of the high esophageal 2-AG levels in humans and experimental EoE, we aimed to explore, the potential of CB_2_ antagonism in inhibiting eosinophil infiltration esophagi of mice. After treating iEoE33 animals with SR 144528 (or vehicle), using a previously published concentration,^35^ for the 14 days of doxycycline exposure, we observed no differences in weight loss or esophageal thickness (Figure 7*A* and *B*). We did, however, observe decreased eosinophil infiltration as determined by flow cytometry (Figure 7*C* and *D*). Given previous studies showing that SR 144528 inhibits ILC2s, we tested whether such results could be also obtained in our iEoE33 mice, especially because eosinophil-ILC2 crosstalk is an important factor in the pathology of allergic diseases.^41^ On gating for ILC2 % (and numbers) (Figure 7*E*), we observed no differences following SR 144528 treatment (Figure 7*F*). In line with previous studies using SR 144528, we noted a significant decrease in CXCL1 mRNA expression in mouse esophagi (Figure 7*G*), whereas there were no differences in the expression of typical T_H_2 markers, such as IL-5 and GATA-3 following SR 144528 application in iEoE33 mice (Figure 7*H* and *I*). Moreover, we observed no effect of SR 144528 treatment on weight loss (Figure 7*J*), esophagus thickness (Figure 7*K*), or eosinophil infiltration (Figure 7*L* and *M*) in esophagi of WT mice.

## Discussion

Despite the importance of the ECS in gut homeostasis, its involvement in EoE pathogenesis has been completely unexplored. We, therefore, focused on investigations of ECS alterations during active EoE inflammation and identified the enzyme MGL to be down-regulated in esophageal mucosal epithelial cells, leading to higher 2-AG levels in patient esophageal mucosal biopsies. Pharmacologic inhibition of MGL in primary human esophageal epithelial cells led to a more “EoE-prone” phenotype expression and increased ability of the epithelial cell secretome to activate and recruit eosinophils via CB_2_ receptors. We validated our findings in vivo and showed that MGL absence similarly resulted in higher eosinophil infiltration, and that specific CB_2_ antagonism reduced eosinophil infiltration in an inducible mouse model of EoE.

In recent years, MGL has emerged as a crucial regulator of 2-AG, involved in many pathophysiological processes, such as stress, metabolic syndrome, pain, and inflammation (reviewed in Grabner et al^5^). MGL is localized to many tissues, and various cell types, showing highest levels in brain, adipose tissue, liver, and intestines.^14^ Physiologically, the interplay of MGL, 2-AG, and CB_1_ is important for the regulation of gut motility.^42^^,^^43^ In TNBS-induced intestinal inflammation, blockade of MGL, an increase in 2-AG, and the activation of CB_1_ and CB_2_ receptors has proven protective, restoring gut homeostasis.^44^ Hence, involvement of MGL in diseases of the esophagus is, therefore, conceivable.

In the human gastrointestinal tract, MGL was previously identified to be expressed in epithelial and immune cells.^14^ Because esophageal epithelial disruption is considered the driver of EoE pathogenesis,^2^^,^^28^ we determined MGL mRNA expression in cytokeratin-positive epithelial cells using ISH method. Our data revealed a decrease in MGL mRNA in epithelial cells during active EoE, which was not observed in other patient groups not characterized by active eosinophilic infiltration. Greuter et al^45^ recently characterized different EoE variants, and in their dataset, MGL was significantly down-regulated only in "classical EoE" and partly in "lymphocytic EoE," whereas gastroesophageal reflux disease and "EoE-like" esophagitis patients showed no alterations in MGL expression (data not shown). In our study, we followed up with publicly available sequencing data, measurements of MGL enzyme activity, and mass spectrometry data of 2-AG levels in patient mucosal esophageal biopsies. Similar to our previous observations in Crohn’s disease^46^ we noticed higher 2-AG levels in active EoE patient mucosal biopsies, and a down-regulation of MGL mRNA in human primary esophageal epithelial cells (H-6046) by IL-13, which was accompanied by an increase in POSTN, a known epithelial gene dysregulated in EoE. This finding is in accordance with previous in vitro data,^47^ altogether suggesting a role for the MGL-2-AG axis and eosinophils in EoE pathology.

Because 2-AG can be rapidly metabolized,^48^ we further investigated whether 2-AG and its action on CB_2_ receptors were responsible for the observed effects on eosinophils. Frei et al^11^ previously described increased CB_2_ surface expression on blood eosinophils of allergic donors. Indeed, we observed that 2-AG acted as a CB_2_-dependent chemoattractant to human eosinophils, which is well in line with Frei et al,^11^ showing that priming of human eosinophils with CB_2_ agonist JWH-133 potentiates migration. Our data, hence, indicate that MGL inhibition in primary esophageal epithelial cells leads to secretome changes characterized by increased 2-AG levels, which are sufficient to potentiate eosinophil migration and activation. An MGL-2-AG-CB_2_ axis, therefore, may not be only a functional entity in the central nervous system,^7^^,^^8^ but also in peripheral tissues.

Absence of MGL in mice has previously been associated with an anti-inflammatory and/or antifibrotic phenotype in the liver.^49^ For the purpose of our study, we explored the consequences of global MGL deletion on healthy esophageal transcriptome, cytokine levels, and eosinophil infiltration in the mouse. Similar to a previous study, we observed higher eosinophil infiltration in healthy MGL KO esophagi as measured by flow cytometry^31^ indicating that eosinophils are primary target cells of the MGL-2-AG axis. Using iEoE33 mice we also validated our patient data by crossing these mice with MGL KO mice. As a result, we detected higher eosinophil infiltration into the esophagi. However, only when performing pharmacologic inhibition of MGL, disease severity in iEoE33 mice was potentiated most likely because MGL depression was already maximal in the iEoE33-MGL KO. A specific CB_2_ inverse agonist reduced the number of infiltrated eosinophils in the mouse esophagi but it remains to be explored whether global CB_2_ KO also dampens EoE severity. In this context, a reduction in BAL eosinophil counts by a CB_2_ inverse agonist was previously reported also in an ILC2-induced model of airway hyperresponsiveness.^12^ However, we did not observe altered ILC2 numbers in the esophagi of our mouse model. Because an orally bioavailable CB_2_ antagonist was recently used in a first phase 1/phase 2 study in humans (NCT05525455), our data prove that antagonistic targeting of CB_2_ receptor may have additional potential for the treatment of allergic diseases characterized by eosinophil infiltration.

Collectively, reduction in eosinophil infiltration by CB_2_ blockade did not ameliorate disease severity, such as weight loss and esophageal thickness, which is in line with previous data showing that iEoE33 mice crossed with eosinophil-deficient mice, still develop the disease.^39^ Moreover, a recent phase 3 clinical study using biologic eosinophil-depleting treatment (benralizumab), while reducing eosinophil counts, did not improve patient symptoms,^50^ corroborating our results. Despite increasing data on eosinophils not being drivers of EoE disease, they still remain an effector cell capable of damaging surrounding tissues and exacerbating disease pathology,^51^ and serving as important diagnostic criteria for EoE.

Our clinical patient data comprising liquid chromatography/mass spectrometry measurements on 2-AG, and MGH enzyme assays, are limited because of the low number of study participants. However, we tried to build on our findings and strengthen our claims by incorporating other publicly available datasets of patients with active EoE into our hypothesis. Although we did observe lower MGL expression in active EoE, the possibility remains this could simply be caused by loss of differentiated epithelial cells, which express most MGL; however, it is very likely that MGL mRNA was down-regulated as shown by our ISH quantification of MGL mRNA signals. In addition, we did not investigate pain symptoms in MGL KO and inhibitor-treated mice. The acute action of MGL inhibitors has been proven to be antinociceptive via an increase in 2-AG acting on CB_1_/CB_2_.^52^ If this were the case, the mice exhibiting less pain might have increased food intake, mitigating weight loss.

## Conclusions

Our study identified dysregulation of the ECS in EoE with a reduction in MGL gene expression and activity, leading to higher 2-AG levels, which aid in eosinophil infiltration into the esophagus via CB_2_ receptors. Given these collective observations, we propose that the ECS plays a role in EoE pathogenesis, and that CB_2_ receptor agonists and phytocannabinoids could increase eosinophil infiltration in the esophagus, potentially exacerbating the disease.

## Materials and Methods

### Patient Characteristics

Age- and sex-matched patients diagnosed with EoE or gastroesophageal reflux disease and control subjects were recruited at the Division of Gastroenterology and Hepatology, University Hospital Graz, from July 2021 to November 2023 (the study was approved by the Institutional Review Board of the Medical University of Graz: clinical study protocol number EK#31-492 ex 18/19; ClinicalTrials.gov, Number: NCT04626609). Two biopsies per patient were immediately fixed in 10% phosphate-buffered formalin for histochemical analysis, and 2 biopsies were snap frozen either for mass spectrometry or MGH activity assays. Biopsies were taken from the distal and the proximal part of the esophagus. Patients with symptomatic and histologically confirmed EoE (≥15 eos/hpf) at the time of sample collection were grouped as “active EoE.” Symptoms of EoE included difficulties swallowing, chest/abdominal pain or heartburn, vomiting, or food impaction. Asymptomatic patients with EoE treated with corticosteroids who had 0 eos/hpf at the time of sample collection were grouped as “EoE in remission.” Control individuals were defined as asymptomatic individuals without a history of esophageal pathologies. Subjects with significant comorbidities, intercurrent illness (eg, infections), and pregnant women were excluded from the study. In total, 12 patients with active EoE, 4 patients with EoE in remission, and 11 asymptomatic control subjects, with no history of esophageal pathologies, were recruited. The complete clinical characteristics of the recruited patients and control subjects are presented in Supplementary Table 4. Patient characteristics have also been published in a previous study.^53^ Blood sampling from healthy volunteers was approved by the Institutional Review Board of the Medical University of Graz (17-291 ex 05/06). All participants provided a written informed consent.

### Animal Work

CB_2_ KO mice (B6.129P2-Cnr2^tm1Dgen^/J on B6 background) were obtained from Jackson Laboratories (Bar Harbor, ME), and then backcrossed for 10 generations. MGL KO mice were a kind gift from Dr R. Zimmermann from the University of Graz,^32^ and bred in-house with WT littermates. The mouse model of inducible EoE, termed iEoE33, was a kind gift from Dr A. Doyle and Dr B. Wright (Mayo Clinic, Arizona, Scottsdale, AZ). In these mice, a secreted and active form of IL-33 is overexpressed in the esophageal epithelium under tetracycline (Tet) inducible expression.^36^^,^^39^^,^^53^ To induce EoE-like pathology, iEoE33 mice and WT control animals were administered doxycycline in water (1 mg/mL in 2% sucrose) for 7 or 14 days. For the duration of the experimental protocol, mice were treated with an MGL inhibitor (JZL 184, 16 mg/kg/d, Cayman Chemical, Ann Arbor) or with a CB_2_ inverse agonist (SR 144528, 10 mg/kg/d, Cayman Chemical). Both JZL 184 and SR 144528 were first dissolved in a mixture of Cremophor and EtOH (1:1) and later dissolved in 5% dextrose water immediately before injection to increase their solubility.^31^^,^^35^

After 7 days, doxycycline was changed to retain its efficacy as described previously.^53^^,^^54^ At the experimental end point (Day 7 or Day 14), total body weight of the mice was measured and their esophagi were harvested for further analysis. Esophagi were weighed and further processed into single-cell suspensions for flow cytometry. Esophageal thickness of different treatment groups was measured with a caliper following esophagus extraction.

Mice were bred and housed in the animal facilities of the Medical University of Graz in accordance with national and international guidelines. All experimental procedures were approved by the Austrian Federal Ministry of Science and Research (protocol number 2021-0.799.497). Male and female mice at 8–12 weeks of age were used in experiments.

### Liquid Chromatography–Mass Spectrometry

2-AG and 1-AG levels were determined in proximal human EoE biopsies and cell culture supernatants using liquid chromatography in combination with tandem mass spectrometry, as previously described by Gurke et al^55^ for analysis of plasma samples. Briefly, 200 μL of tissue homogenate or 800 μL of supernatant (a higher sample volume was used because of lower analyte concentration) were extracted by liquid-liquid extraction after the addition of the internal standard with 400 μL or 1600 μL of a mixture of ethyl acetate/hexane (9:1 vol/vol). The organic phase was evaporated and then resuspended in 50 μL of acetonitrile. The subsequent liquid chromatography/mass spectrometry analysis was performed on a QTRAP 6500+ triple quadrupole mass spectrometer (Sciex, Darmstadt, Germany) equipped with a Turbo Spray ion source operated in positive electrospray ionization mode and coupled to an Agilent 1290 Infinity II UHPLC system (Agilent, Waldbronn, Germany). Chromatographic separation was achieved using a binary gradient on an Acquity UPLC BEH C18 column (100 × 2.1 mm, 1.7 μm; Waters, Eschborn, Germany) with a precolumn of the same type. A solution of 0.0025% formic acid in water was used as solvent A, and 0.0025% formic acid in acetonitrile was used as solvent B, with a total run time of 8 minutes.

### MGH Activity Assay

MGH was determined in lysates prepared from snap-frozen EoE patient biopsies or mouse esophageal tissues as described previously with some modifications.^32^ In brief, tissues were washed and homogenized on ice in lysis buffer using an Ultra Turrax (IKA, Staufen, Germany). Lysates were centrifuged at 10.000 × *g* for 10 minutes at 4°C, and the cytoplasmic fraction was used to measure MGH activity. Protein concentrations of tissue lysates were determined using the Bio-Rad protein assay according to the manufacturer's instructions (Bio-Rad) and bovine serum albumin as the standard. Depending on the tissue, 20–50 μg of protein was incubated with 100 μL of MG substrate (*1,3-rac*-oleoylglycerol, 1 mM) for 30 minutes at 37°C. Thereafter, the reaction was stopped by the addition of 100 μL of chloroform and vortexing. Phases were separated by centrifugation at 10,000 × *g* for 5 minutes at 4°C. Glycerol concentrations in the aqueous phase were determined using a commercial kit (free glycerol reagent, Sigma).

### Histology

Human esophageal mucosal biopsies and mouse esophagus samples were immediately fixed in 10% phosphate-buffered formalin for histochemical analysis, and left for 24–48 hours at RT with gentle shaking. Tissue was processed and embedded in paraffin, according to standard procedures as described previously.^31^^,^^35^

### ISH and Immunofluorescence

Tissue was cut in 5-μm sections, baked at 60°C for 1 hour, dewaxed, and rehydrated. ISH was performed according to the manufacturer’s protocol and as recently published.^35^ Sirius red (Direct Red 80, Sigma) was used to stain eosinophils in deparaffinized sections. ISH probes used to detect MGL and CB_2_ mRNA were purchased from Advanced Cell Diagnostics (Newark). ISH was performed using the RNAscope 2.5 HD red kit according to manufacturer instructions. Immunofluorescence of epithelial and immune cells in esophageal tissue was performed with an anti-cytokeratin primary antibody (1:200; Dako #ZO0622), an anti-EPX antibody, or a biotinylated anti-EPX antibody (kind gifts from Elizabeth Jacobsen, Mayo Clinic Arizona, Scottsdale, AZ). Alexa Fluor 488-labeled goat anti-rabbit IgG (1:500, Jackson Immuno Research, #111-546-144) was used as the secondary antibody. In parallel, sections were processed in the absence of primary antibodies as negative controls. The sections were then mounted with Vectashield (containing DAPI) (Vector Laboratories) and images were taken using an Olympus IX73 fluorescence microscope (Olympus), connected to a Hamamatsu ORCA-ER digital camera (Hamamatsu Photonics K.K., Japan). Images were processed with an Olympus CellSens 1.17 imaging software (Olympus). ImageJ was used for the analysis. Only image contrast and brightness were adjusted.

### Primary Esophageal Epithelial Cells

Human primary esophageal epithelial cells (#H-6046) were acquired from CellBiologics (Chicago). Cells were cultivated in flasks precoated with a 0.1% gelatin solution as previously described.^53^ Human epithelial cell medium (CellBiologics #H6621) supplemented with 5% fetal bovine serum, 1% antibiotic-antimycotic solution, 0.1% hydrocortisone, and 0.1% epidermal growth factor was used to grow the cells at 37°C and 5% CO_2_ in a humidified atmosphere. Before experimental treatment, cells were seeded in 6-well plates and on reaching 80%–90% confluency, starved for 4–24 hours in human epithelial cell medium without fetal bovine serum or supplements. After the starvation period, cells were treated either with vehicle, MGL inhibitors JZL 184 and MJN 110 (both Cayman Chemical), or recombinant human IL-13 (BioLegend, San Diego) for 24 hours. Following the incubation period, supernatants were collected, centrifuged to remove potential cell debris, and stored at -80°C until analysis. Cells were collected using TRIzol and stored at -80°C until RNA extraction.

### Eosinophil Isolation

Polymorphonuclear leukocyte preparations were isolated from healthy individuals by density gradient centrifugation (Lympho Spin Medium, pluriSelect) as previously described.^53^ First, platelet-rich plasma was removed via centrifugation. Next, red blood cells and platelets were removed via dextran sedimentation, and polymorphonuclear leukocytes preparations were obtained via density gradient separation. Eosinophils were isolated from polymorphonuclear leukocytes via negative magnetic selection using an Eosinophil Isolation Kit (Miltenyi Biotec, Bergisch Gladbach, Germany). Eosinophil purity was determined by morphologic analysis of Kimura-stained cells, and was typically greater than 97%.

### Shape Change Assay

The eosinophil shape change assay following supernatant incubation was performed as described previously,^56^ where shape change was determined as the increase in the forward scatter (FSC) property of the cell. Approximately 5 × 10^4^ of isolated eosinophils per sample were suspended in assay buffer containing Ca^2+^ and Mg^2+^, preincubated with epithelial cell supernatants at a ratio of 1:3 (30 minutes, RT), and then stimulated (4 minutes, 37°C) with human eotaxin-1 (CCL11). Afterward, cells were transferred onto ice, and ice-cold fixative solution was added to terminate the reaction and maintain the change in cell shape until analysis. The samples were analyzed on a FACS Canto II flow cytometer (Becton Dickinson, Mountain View, CA), where shape change was determined as the increase in the forward scatter (FSC) property of the cell, and was normalized to the unstimulated vehicle control.

### Migration Assay

Purified human eosinophils were pretreated with either vehicle or CB_2_ inverse agonist SR 144528 (10 μM, 30 minutes, RT), and they were allowed to migrate to the epithelial cell supernatants or 2-AG at different concentrations in an HTS Transwell 96-well plate with a 5-μm pore size polycarbonate membrane (1 hour, 37°C).^56^

Mouse splenocytes were isolated from the spleen of WT C57BL/6J and CB_2_ KO mice by meshing tissue through a 40-μm strainer. Isolated splenocytes were lysed of red blood cells (eBiosience #00433357) and counted. Afterward, cells were stained with surface FC Panel 1 (Supplementary Table 1). After staining, cells were washed and resuspended in assay buffer. 2 x 10^5^ cells per sample were added to each well of a HTS Transwell 96-well system with a 3-μm pore size. Different concentrations of the specific CB_2_ agonist JWH-133 (10 nM–1 μM), or of 2-AG (10 nM–1 μM, Cayman Chemicals #62160) were added to the bottom compartment. The cells were left to migrate for 2–4 hours at 37°C and 5% CO_2_. After migration, cells were fixed and transferred to 1.1 ml Micro Tubes (Bioquote, York, UK, #TN0946-01B) for measuring.

Human eosinophils that had migrated to the lower compartment were counted for 1 minute by flow cytometric counting on a FACS Canto II (Becton Dickinson).^57^ Mouse splenocytes that migrated to the lower compartment were counted for 1 minute by a BD LSR Fortessa flow cytometer. Mouse eosinophils were identified as viable, single, CD45^+^, CD3^-^, Siglec-F^+^, and high SSC cells.

The number of migrated cells was determined by flow cytometry, and the chemotactic index was calculated as the fold change of migrated cells compared with the vehicle control (no chemoattractant). In the case of migration toward epithelial cell supernatants, the number of migrated cells was normalized to vehicle-treated epithelial cell supernatant.

### RNA Extraction and Reverse Transcription Quantitative Polymerase Chain Reaction

Mouse esophageal tissue and epithelial cell samples were stored at -80°C until RNA extraction with TRIzol (Invitrogen by Thermo Fisher Science #11596018) was performed. Samples were treated with a DNA-free DNA Removal Kit (Invitrogen). The quality and concentration of RNA were determined using a NanoDrop ND-1000 spectrophotometer (Thermo Fisher Scientific). Reverse transcription of purified RNA was performed using a High-Capacity cDNA Reverse Transcription Kit (Applied Biosystems). Gene expression was assessed by reverse transcription-quantitative polymerase chain reaction using SsoAdvanced Universal SYBR Green Supermix (Bio-Rad). Primers were acquired from Eurofins (Supplementary Table 5) and Bio-Rad (for human MGL, IL-33, COX-2, and HPRT).

### Single-Cell Suspensions and Flow Cytometric Phenotyping of Immune Cells

Weighed mouse esophagi (minimum 15 mg of weight) were cut into small pieces and then digested with DNase I (160 U/mL; Worthington) and collagenase (4.5 U/mL; Worthington) for 20 minutes at 37°C, while rotating at 750 rpm as described previously.^53^ After that, the tissue was passed through a 40-μm strainer. Samples were then resuspended in staining buffer (phosphate-buffered saline + 2% fetal bovine serum), washed with phosphate-buffered saline, and used for surface and antigen staining. Immediately after digestion, 20 μL of counting beads (Precision Count Beads, BioLegend) were added to each sample.

To exclude dead cells, single-cell suspensions from tissue or splenocytes were initially incubated for 20 minutes in Fixable Viability Dye eFluor 780 (eBioscience) in phosphate-buffered saline at 4°C in the dark. Before staining, the single cell suspensions were incubated in 1 μg anti-mouse or anti-human TruStain FcX (BioLegend, #422304 or #101320, RRID: AB 1574975) for 10 minutes. Immunostaining was performed for 30 minutes at 4°C (protected from light) using a premixed antibody panel (Supplementary Tables 1, 3, and 6). Cells were washed and fixed in eBioscience IC Fixation Buffer (Thermo Fisher Scientific, #00-8222-49) for 10 minutes at 4°C. Cells were finally washed and resuspended in staining buffer to be acquired on a BD LSR Fortessa flow cytometer with FACSDiva software (BD Biosciences) within 3 days of the experiment. FlowJo software (Treestar) was used for analysis and compensation. Fluorescence minus-1-samples were used to define the gates of cell populations and activation markers (see gating strategy in Figures 6*A* and 7*E*).

### Multiplex Enzyme-Linked Immunosorbent Assay

Cytokine concentrations in snap-frozen pieces of extracted esophagi from WT and MGL KO mice were evaluated using the custom ProcartaPlex immunoassay (eBioscience) according to the manufacturer’s specifications. Fluorescent signals were quantified with the Bio-Plex 200 multiplex suspension array system equipped with Luminex xMAP technology combined with the Bio-Plex 5.0 software (Bio-Rad, Hercules) as described previously.^56^

### RNAseq

RNA was isolated from frozen mouse esophagus tissues of WT and MGL KO mice and analyzed using GENEWIZ (Azenta Life Sciences). rRNA was removed by PolyA selection. The total RNA was sequenced on an Illumina NovaSeq (6000 and the X Plus), and 20 million reads per sample were acquired. Quality control was performed with fastqc (0.11.9), adapter trimming with Cutadapt and sequences were aligned using STAR (2.7.10b) against the mouse reference genome (GRCm39). Subsequent differential gene expression analysis was done in RStudio 4.1.3 using the edgeR package (version 3.36.0) and pathway enrichment analysis was performed using pathfindR (version 2.3.1). All RNAseq data analysis was done in RStudio (version 4.4.1). For differential expression testing, edgeR (version 2.4.0) was used, the data normalized and the exact test was performed. The volcano plot was generated with R package ggplot2. RNAseq data are deposited at GEO database, accession number GSE282590.

### Statistical Analysis

Data are presented as means + standard error of means unless otherwise indicated. Statistical analyses for experiments was performed using GraphPad Prism 10.0.3 (GraphPad Software). Differences between 2 experimental groups were evaluated using unpaired or paired Student *t*-tests, whereas differences between multiple experimental groups were assessed using 1-way or 2-way analysis of variance. In all cases, a *P* < .05 was considered significant. Publicly available total transcriptome data were acquired from EGID Express https://egidexpress.research.cchmc.org/data/ (accessed on August, 2024).
