# Supplementary material for: The Endocannabinoid System Drives Eosinophil Infiltration During Eosinophilic Esophagitis
Source: Cell Mol Gastroenterol Hepatol. 2025 Apr 11;19(8):101515. doi: 10.1016/j.jcmgh.2025.101515 (PMC12143766; doi:10.1016/j.jcmgh.2025.101515)
Supplement: Supplementary Material [file mmc1.pdf]

Supplementary Tables

Supplementary Table 1: FC Panel 1 for in vitro migration of splenocytes.

Antibody	Dilution	Clone	Company	Catalogue #	RRID
FVD ef780	1:2000	/	eBioscience	65-0865-18	AB_2869673
CD45-AF700	1:400	30-F11	Biolegend	103128	AB_493715
CD3-BUV395	1:80	145-2C11	BD	563565	AB_2738278
CD4-BUV496	1:160	GK1	BD	564667	AB_2722549
CD8-PerCPCy5.5	1:160	53-6.7	Biolegend	100734	AB_2075239
Siglec-F-PE	1:80	1RNM44N	BD	562068	AB_10896143

Supplementary Table 2: Significantly differentially expressed genes between WT and MGL KO mouse esophagi.

Gene	logFC	logCPM	PValue	fdr
Kbtbd12	-2,97151124	5,775166095	1,12228E-72	3,68657E-68
Mgll	2,626324518	5,921551938	3,29023E-36	5,40404E-32
Dusp1	-1,74648655	6,689146416	7,813E-16	8,55497E-12
Ccn1	-1,534761112	4,310839018	4,76884E-12	3,91629E-08
Myh3	-2,040338462	4,586043239	3,63508E-10	2,38817E-06
Slc25a25	-1,211599144	4,613750251	1,96266E-09	1,07452E-05
Fos	-2,691813417	4,665791944	7,37887E-09	3,46269E-05
Pmepa1	-0,919315465	5,5275946	1,58138E-08	6,49335E-05
Serpina3n	2,365373465	6,165125601	8,28436E-08	0,00030237
Ccn2	-1,503794836	5,463973797	3,55699E-07	0,001168436
Gm21451	7,015076434	-0,352704926	4,88507E-07	0,001458816
Atcay	1,834740263	4,613529046	6,3151E-07	0,00162689
Nfil3	-1,767502802	3,750481727	6,43842E-07	0,00162689
Cirbp	0,789443342	6,256987466	1,54354E-06	0,003621701
Myl10	-1,579082344	3,86044908	3,002E-06	0,006574175
Epyc	2,270637044	1,321922644	4,99424E-06	0,010253491
Dysf	-0,915305831	7,309695157	6,70494E-06	0,012054808
Ltf	-9,481628753	7,504392307	6,97255E-06	0,012054808
Tnfrsf12a	-1,039347495	4,682412283	6,77065E-06	0,012054808
Plac9	1,623082656	6,312897816	1,18735E-05	0,019501679
Lyl1	1,270873071	3,293959617	2,21637E-05	0,034669358
Cebpd	-1,116980282	5,165633312	2,74073E-05	0,04092286
Cdkn1a	-0,689994836	7,815606202	2,92452E-05	0,041768475
Gadd45g	-1,042177683	6,284387898	3,17969E-05	0,042925762
Lrrc23	-6,406982508	2,639979952	3,36744E-05	0,042925762
Ttc21a	-7,336450973	3,171060352	3,39758E-05	0,042925762
Dio2	-1,217120206	2,679939228	3,84092E-05	0,046729762

Supplementary Table 3: FC Panel 2 for eosinophil determination in single cell suspensions in mouse esophagi.

Antibody	Dilution	Clone	Company	Catalogue #	RRID
FVD ef780	1:2000	/	eBioscience	65-0865-18	AB_2869673
CD45-AF700	1:400	30-F11	Biolegend	103128	AB_493715
CD3-PE-Cy7	1:50	145-2C11	eBioscience	25-0031-82	AB_469572
CD4-BUV496	1:160	GK1	BD	564667	AB_2722549
CD8-PerCPCy5.5	1:160	53-6.7	Biolegend	100734	AB_2075239
Siglec-F-PE	1:80	1RNM44N	BD	562068	AB_10896143
NKp46-BV510	1:20	29A1.4	Biolegend	137623	AB_2563290
CD11b-BUV737	1:80	M1/70	BD	612801	AB_2870128
CD19-BV650	1:30	6D5	Biolegend	115541	AB_11204087
Ly6G-PEDazzle594	1:166	1A8	Biolegend	127648	AB_2566319
PD1-BV421	1:80	29F.1A12	Biolegend	135221	AB_2562568

Supplementary Table 4: Patient clinical characteristics. Data are represented as median and interquartile range or percentage when indicated.

		Controls	Active EoE	Inactive EoE
**N**		11	12	4
**Age (y)**		58<br>(52,69)	45.5<br>(31,52)	52.5<br>(47.8, 56.5)
**Gender male n (%)**		45	75	100
**Eosinophils / hpf**		0	27.5<br>(25,50)	0
**Treatment (%)**	PPI	9	/	/
	Corticosteroid (topical)	/	33	100
	NSAID	9	/	/

Supplementary Table 5: Eurofin Genomics Primer sequences used for RT-qPCR.

Species	Target	Sequence	Direction
**human**	SPINK7	5´-GCTCAGAAGCTGCTAGTCTGT-3´	fwd
		5´-GCTCTCGGTACACAAGTGACA-3´	rev
	POSTN	5´-AGATCCGTGAAGGTGGTTTG-3´	fwd
		5´-GTCTTTGAGACGCTGGAAGG-3´	rev
	DSG1	5´-ATGTCTGCAGAGAGCGAGTG-3´	fwd
		5´-CCAATTTCCCTCATTTCCAG-3´	rev
	IL-13	5´- GCAATGGCAGCATGGTATGG -3´	fwd
		5´- AAGGAATTTTACCCCTCCCTAACC-3´	rev
**mouse**	IL-13	5´-AGA CCA GAC TTC CCT GTG CA-3´	fwd
		5´-TGG GTG CTG TAG ATG GCA TTG-3´	rv
	CCL11	5´-AGA CCA GAC TCC CCT GTG CA-3´	fwd
		5´-TGG GTC CTG TAG AGT GCA TTG-3´	rv
	HPRT	5´-TCA GTC AAC GGG GGA CAT AAA-3´	fwd
		5´-GGG GCT GTA CTG CTT AAC CAG-3´	rv
	IL-5	5´-GCT GGC CTC AAA CTG GTA ATG TA-3´	fwd
		5´-GGC AAT GGT TGC AGT TGT GAT ACC-3´	rv
	GATA-3	5´-AAG AAA GGC ATG AAG GAC GC-3´	fwd
		5´-GTG TGC CCA TTT GGA CAT CA-3´	rv
	CXCL-1	5´-CCA AAC CGA AGT CAT AGC CAC A-3´	fwd
		5´-GAG GTG CCA TCA GAG CAG TC-3´	rv

Supplementary Table 6: FC Panel 3 for ILC2 determination in single cell suspensions in mouse esophagi.

<table>
<tr><th colspan="2">Antibody</th><th>Dilution</th><th>Clone</th><th>Company</th><th>Catalogue #</th><th>RRID</th></tr>
<tr><td colspan="2">FVD ef780</td><td>1:2000</td><td>/</td><td>eBioscience</td><td>65-0865-18</td><td>AB_2869673</td></tr>
<tr><td colspan="2">CD45-BV785</td><td>1:350</td><td>30-F11</td><td>Biolegend</td><td>103149</td><td>AB_2564590</td></tr>
<tr><td colspan="2">CD90.2 -eFluor450</td><td>1:200</td><td>53-2.1</td><td>eBioscience</td><td>48-0902-82</td><td>AB_1272200</td></tr>
<tr><td colspan="2">Ly-6A/E-BV711</td><td>1:100</td><td>D7</td><td>BD</td><td>563992</td><td>AB_2738529</td></tr>
<tr><td colspan="2">IL33R (ST2)-PE</td><td>1:100</td><td>U29-93</td><td>BD</td><td>566312</td><td>AB_2744490</td></tr>
<tr><td colspan="2">KLRG1-PErCP-Cy5.5</td><td>1:100</td><td>2F1</td><td>BD</td><td>563595</td><td>AB_2738301</td></tr>
<tr><td>Ter-119-PE-Cy7</td><td rowspan="11">Lineage</td><td>1:75</td><td>TER-119</td><td>eBioscience</td><td>25-5921-82</td><td>AB_469661</td></tr>
<tr><td>B220-PE-Cy7</td><td>1:200</td><td>RA3-6B2</td><td>eBioscience</td><td>25-0452-82</td><td>AB_469627</td></tr>
<tr><td>CD3-PE-Cy7</td><td>1:100</td><td>145-2C11</td><td>eBioscience</td><td>25-0031-82</td><td>AB_469572</td></tr>
<tr><td>CD11c-PE-Cy7</td><td>1:100</td><td>HL3</td><td>BD</td><td>558079</td><td>AB_647251</td></tr>
<tr><td>CD11b-PE-Cy7</td><td>1:150</td><td>M1/70</td><td>eBioscience</td><td>25-0112-82</td><td>AB_469588</td></tr>
<tr><td>CD19-PE-Cy7</td><td>1:75</td><td>1D3</td><td>eBioscience</td><td>25-0193-82</td><td>AB_657663</td></tr>
<tr><td>Gr1-PE-Cy7</td><td>1:200</td><td>RB6-8C5</td><td>eBioscience</td><td>25-5931-82</td><td>AB_469663</td></tr>
<tr><td>FcƐR1α-PE-Cy7</td><td>1:100</td><td>MAR-1</td><td>eBioscience</td><td>25-5898-82</td><td>AB_2573493</td></tr>
<tr><td>CD4-PE-Cy7</td><td>1:200</td><td>GK1.5</td><td>eBioscience</td><td>25-0041-82</td><td>AB_469576</td></tr>
<tr><td>CD8-PE-Cy7</td><td>1:100</td><td>53-6.7</td><td>BD</td><td>552877</td><td>AB_394506</td></tr>
<tr><td>NK1.1-PE-Cy7</td><td>1:100</td><td>PK136</td><td>eBioscience</td><td>25-5941-82</td><td>AB_469665</td></tr>
</table>
